# Noninvasive Determination of the IDH Status of Gliomas Using MRI and MRI-Based Radiomics: Impact on Diagnosis and Prognosis

**DOI:** 10.3390/curroncol29100542

**Published:** 2022-09-23

**Authors:** Yurong Li, Qin Qin, Yumeng Zhang, Yuandong Cao

**Affiliations:** 1 Department of Radiation Oncology, Nanjing Medical University First Affiliated Hospital, Nanjing 210029, China; 2 The First School of Clinical Medicine, Nanjing Medical University, Nanjing 210029, China

**Keywords:** glioma, IDH1 mutation, magnetic resonance imaging, radiomics, prognosis

## Abstract

Gliomas are the most common primary malignant brain tumors in adults. The fifth edition of the WHO Classification of Tumors of the Central Nervous System, published in 2021, provided molecular and practical approaches to CNS tumor taxonomy. Currently, molecular features are essential for differentiating the histological subtypes of gliomas, and recent studies have emphasized the importance of isocitrate dehydrogenase (IDH) mutations in stratifying biologically distinct subgroups of gliomas. IDH plays a significant role in gliomagenesis, and the association of IDH status with prognosis is very clear. Recently, there has been much progress in conventional MR imaging (cMRI), advanced MR imaging (aMRI), and radiomics, which are widely used in the study of gliomas. These advances have resulted in an improved correlation between MR signs and IDH mutation status, which will complement the prediction of the IDH phenotype. Although imaging cannot currently substitute for genetic tests, imaging findings have shown promising signs of diagnosing glioma subtypes and evaluating the efficacy and prognosis of individualized molecular targeted therapy. This review focuses on the correlation between MRI and MRI-based radiomics and IDH gene-phenotype prediction, discussing the value and application of these techniques in the diagnosis and evaluation of the prognosis of gliomas.

## 1. Introduction

Gliomas are the most common primary malignant brain tumors in adults, with an annual incidence of 6/100,000 [1]. Gliomas account for almost 80% of all malignant brain tumors and are responsible for the majority of brain tumor-related deaths [2]. The 2007 WHO Classification of Tumors of the Central Nervous System classified gliomas into grades I to IV according to histology, with the main classifications being astrocytomas, oligodendrogliomas, and ependymas [3]. In 2016, the WHO Classification first added molecular parameters to the previous classifications to define tumor entities [4], improving the accuracy of prognosis prediction and guiding individualized treatment. The fifth edition of the WHO Classification of Tumors of the Central Nervous System, published in 2021, building on the 2016 update, provided molecular and practical methods for the central nervous system (CNS) tumor taxonomy. The fifth edition introduced major changes that advance the role of molecular diagnostics in CNS tumor classification [5]. Among them, isocitrate dehydrogenase (IDH) has been one of the most studied.

**Figure 1:**
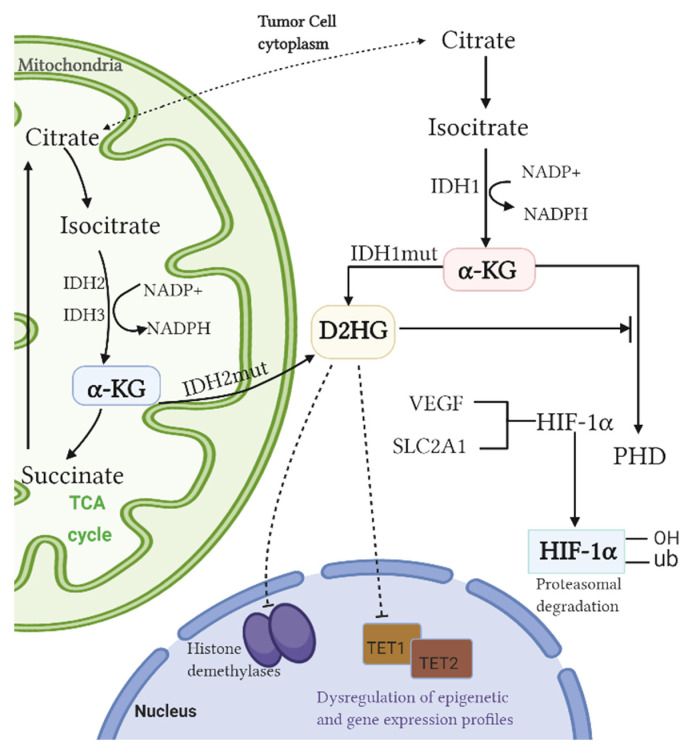
Mechanism by which IDH mutation promotes tumorigenesis.

In 2008, Parsons et al. first found IDH1 mutant somatic cells in glioblastoma (GBM) patients through genome-wide sequencing [6]. IDH1 and IDH2 mutations were subsequently found in patients with WHO grade II/III gliomas [7,8]. IDH plays a unique role in cells, catalyzing the oxidative decarboxylation of isocitrate to a-ketoglutarate (a-KG). Mutated IDH1 (cytoplasmic peroxidase) and IDH2 (mitochondria) [9] almost completely lose this ability, resulting in a decrease in α-KG-dependent prolyl hydroxylase (PHD) activity, which directly increases HIF-1α expression [10]. HIF-1α is a transcription factor involved in metabolism, angiogenesis, and tumorigenesis. The overexpression of HIF-1α is associated with poor prognosis and the progression of multiple cancers [11]. Apart from a loss of normal catalytic activity, mutant IDH also has new enzymatic activity, causing the reduction of α-KG to D-2-hydroxy glutaric acid (D-2HG). D-2HG and α-KG share a similar architecture, resulting in the binding of D-2-hydroxy and α-KG-dependent dioxygenases (histone demethylases [12], 5-methylcytosine hydroxylases of the TET family [13], etc.), and act as competitive inhibitors. These changes increase histone methylation and decrease 5-hydroxymethylcytosine on a genome-wide scale. Histone and DNA methylation related to IDH mutations may promote tumorigenesis by altering epigenetic and gene expression profiles [14] (Figure 1).

Mutant IDH can lead to longer overall survival and is an essential and relatively independent prognostic factor [7,15]. Pretreatment identification of IDH status can lead to further clinical decisions, early intervention, and better management for tumor patients. Currently, glioma treatment consists of surgical resection, chemotherapy, and radiation [16]. Immunohistochemistry or gene sequencing of tumor tissue is the standard approach to identify mutant genes. Tumor tissue can be obtained by surgery or tissue biopsy, and although the procedure is usually safe, the risks associated with the procedure may be significant, especially in brain cancer [17]. Due to regional heterogeneity, surgical risks are further amplified in situations where serial biopsies are required [18]. Additionally, the challenges related to intratumoral heterogeneity [19,20], sampling errors [21], and biopsy timeliness [22,23] present formidable obstacles to surgical planning. Noninvasive models such as MRI and MRI-based radiomics imaging are shown to be potential tools that can provide pathogenesis insights and benefit diagnostic processes, therapeutic responses, and follow-up [24,25]. In this article, we focus on recently published studies, discussing the value and applications of MRI and MRI-based radiomics in the diagnosis and evaluation of the prognosis of gliomas.

## 2. Correlation of Conventional Magnetic Resonance Imaging (cMRI) Findings with IDH Mutation Status and The Prognosis of Gliomas

### 2.1. Location

The anatomic position of glioma may determine tumor resectability and affect the treatment and prognosis. The site of a tumor can be clearly shown in cMRI. Several studies have found that tumors with IDH mutations rarely grow in high-risk regions but instead in functional or nonfunctional areas, especially the frontal lobe and temporal lobe [26,27,28] (Table 1). Song et al. analyzed the gene-phenotype of 193 astrocytomas and found that IDH-mutated gliomas were mainly located in a single lobe, such as the temporal lobe, frontal lobe, or cerebellum. IDH-wildtype tumors were located in combined lobes, such as the brainstem or diencephalon (*p* < 0.001) [26] (Table 1). These observations are similar to those of previous studies [29], possibly because neuroglial progenitor cells in the subventricular zone likely give birth to cells of IDH-mutated gliomas [30,31]. Different types of gliomas may arise from different precursor cells that are relatively region-specific at inception or during brain development. It is well documented that IDH1-mutated gliomas arise from a distinct ‘cell of origin’. Studies suggest that ‘cells of origin’ for IDH1-mutated gliomas may exist as part of a neural precursor population with limited differentiation potential that is mostly confined to the frontal lobe, specifically to the area surrounding the rostral extension of the lateral ventricles, provided supporting radiological evidence for this hypothesis [28,32].

### 2.2. Enhancement

**Figure 2:**
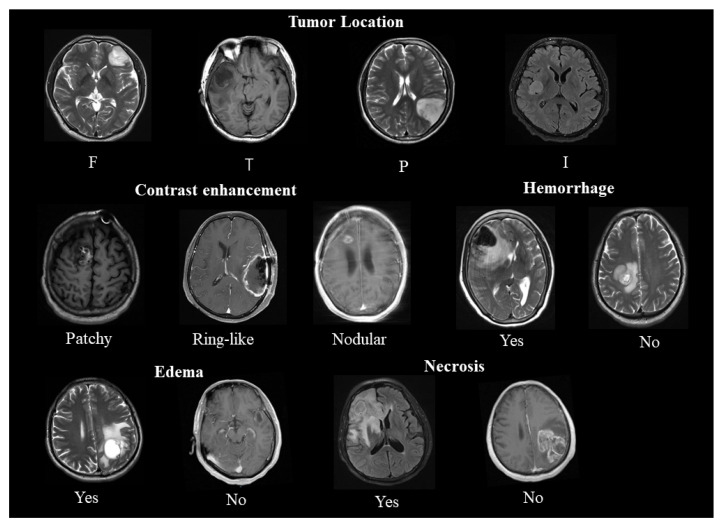
A representative example of conventional magnetic resonance imaging features. *Upper panel:* tumor location (F: frontal, T: temporal, P: parietal, I: insular). *Second panel:* patterns of contrast enhancement (**left**) and hemorrhage (**right**). *Lower panel:* edema (**left**) and necrosis (**right**).

Blood–brain barrier disruption-based pathophysiological changes are the main reason for contrast enhancement (CE) in MR images [33] (Figure 2). Multiple studies have shown that enhancement was more common in IDH-wildtype gliomas than in IDH-mutated gliomas [33,34] (Table 1). Notably, the median survival was 780 days for GBM patients with nCET (defined as: demonstrated any amount of non-enhancing solid tissue) compared with 465 days without (*p* < 0.02), suggesting that IDH mutations may reflect a higher malignant potential [35]. The expression of angiogenesis genes, which is positively correlated with the presence of vascular permeability [36], was upregulated in contrast enhancement regions [37,38], resulting in increased contrast. However, IDH-mutant gliomas had fewer enhanced regions, suggesting lower vascular endothelial growth factor (VEGF) levels, which is contrary to previous reports [39]. Interestingly, Suchorska et al. found that only in IDH-mutated tumors was CE associated with lower survival rates (*p* < 0.005), while prognosis in IDH-wildtype tumors was independent of CE (*p* = 0.31) [40]. Overall, IDH-wildtype gliomas are more likely to have enhancement than IDH-mutated gliomas, and the degree of malignancy correlates with a relatively worse prognosis. Moreover, the patterns of contrast enhancement may be more useful for outcome stratification and prognosis estimation in patients with IDH-mutated gliomas than for stratifying PFS and OS in patients with IDH-wildtype gliomas [33]. Voss et al. also found that new contrast-enhancing spots (NCEs) were common in young patients with IDH-mutated gliomas after radiotherapy [41], which may be related to the vascular lesions, hypoxia, and tissue necrosis caused by radiotherapy [42]. Notably, of the 23 patients whom Voss et al. followed up, 11 NCEs disappeared spontaneously after 3 years, and 7 NCEs remained untreated but stable, suggesting a relatively good prognosis [41].

### 2.3. Edema, Necrosis, and Hemorrhage

Several studies have shown that edema, necrosis, and hemorrhage are associated with poor prognosis. Tumor-induced edema, an inflammatory response, was a prognostic factor for patients with MGMT promoter methylation [43]. Pope et al. found a median survival of 442 days in patients with edema and 1098 days in patients without edema (*p* < 0.002) [40]. Notably, edema was inversely associated with NCET, with younger patients having less edema and more NCET [35]. This may explain why younger patients tend to have longer median survival. Conversely, Wang et al. indicated that in the mutant IDH1 group, the absence of edema predicted longer OS (*p* = 0.032) and PFS (*p* = 0.024), and there were no remarkable differences in edema between glioma patients with mutant (13/45, 28.9%) and IDH1-wildtype (54/235, 23.0%) (*p* = 0.395, chi-square test) [34] (Table 1). The median survival time of patients with necrotic anaplastic gliomas (n = 8) was 443 days (mean, 816 days; SE, 197 days) and that of patients with nonnecrotic anaplastic gliomas (n = 31) was 773 days (mean, 2270 days; SE, 246 days) [34]. Patients with IDH1-wildtype had a hemorrhage tendency compared to patients with mutant IDH1 (15 vs. 5; *p* = 0.286) [44].

## 3. Correlation of Advanced Magnetic Resonance Imaging (aMRI) Findings with IDH Mutation Status and The Prognosis of Gliomas

In the absence of CE, necrosis, and edema, cMRI may misdiagnose high-grade gliomas (HGGs) as low-grade gliomas (LGGs). aMRI (DWI, PWI, MRS, etc.), now widely used in the preoperative evaluation and follow-up of gliomas, provides information (angiogenesis, blood volume, micronecrosis, cellular information, etc.) that is important to determine tumor grades but cannot be provided by cMRI.

### 3.1. Perfusion Weighted Imaging (PWI)

Previous research has revealed that IDH-mutated gliomas can reduce the activation of HIF-1α, leading to the inhibition of angiogenesis and related signals [45]. Blood perfusion characteristic analysis can help in the evaluation of the prognosis of glioma patients with different IDH statuses. Dynamic susceptibility contrast-perfusion weighted imaging (DSC-PWI), with the measurement of the relative cerebral blood volume (rCBV), may yield noninvasive information on tumor microvessels [46]. Several reports have shown that there is a notable difference in the rCBV value in the enhanced region between IDH-mutated and IDH-wildtype tumors, and the rCBV_max_ is significantly correlated with IDH-mutated tumors [47,48]. Xing et al. found that the rCBV_max_ of IDH-mutated glioblastomas was significantly lower than that of IDH-wildtype glioblastomas (IDH-mutated = 5.08 ± 1.48; IDH-wildtype = 8.93 ± 2.99; *p* < 0.001) [49]. This finding corresponds to that of their previous study (Xing et al., 2017) of 42 astrocytomas, in which the rCBV_max_ (1.41 ± 0.50) of IDH-mutated gliomas was significantly lower than that of IDH-wildtype gliomas (3.47 ± 2.34) (*p* = 0.004) [50] (Table 2). That is, IDH-wildtype tumors are associated with a considerably higher rCBV [46]. Some research also indicated that patients with complete remission and stable disease had a lower rCBV than those with progressive disease and death [51]. The rCBV has tremendous evaluation potential in the angiogenesis of tumors with different IDH phenotypes, with higher values being associated with increased vascular proliferation and neovascularization.

### 3.2. Diffusion Weighted Imaging (DWI)

As a quantitative imaging method, DWI can noninvasively observe the Brownian motion of water and reflect the cellular architecture through the apparent diffusion coefficient (ADC). Tan et al. reported that a relatively less apparent diffusion coefficient (rADC_min_) and relatively more apparent diffusion coefficient (rADC) may be used to identify whether IDH is mutated [52]. The rADC has emerged as a valuable adjunct in glioma genotype and prognosis studies [53,54]. Hong et al. also reported that the average ADC_T2-T1_ (hyperintensity of the T2WI necrotic or cystic region—contrast-enhancing region volume) had the strongest correlation with IDH mutation status [47]. A study compared the ADCs of astrocytoma patients and found that the rADC (IDH-mutated = 1.88 ± 0.41; IDH-wildtype = 1.37 ± 0.31; *p* < 0.001) and ADC_min_ (IDH-mutated = 1.21 ± 0.27, IDH-wildtype = 0.87 ± 0.18; *p* < 0.001) of IDH-mutated astrocytomas were significantly higher than those of wildtype astrocytomas [50] (Table 2). Xing et al. proposed that when the rADC_min_ of a GBM was >0.98, it was suggestive of an IDH-mutated GBM [49]. These results indicate that IDH-wildtype tumor cells are relatively dense, and the limitation of extracellular water molecule diffusion is more obvious. IDH-wildtype GBMs are speculated to have a higher malignant degree and a relatively poorer prognosis than IDH-mutated GBMs. As demonstrated by Feraco et al., IDH-mutated astrocytomas showed a much higher ADC than IDH-wildtype astrocytomas, and a positive association between the ADC_mean_ and OS in the overall group was also identified (*p* = 0.003; R = 0.62) [55].

### 3.3. Diffusion Tensor Imaging (DTI)

DTI is an extension of DWI. DTI can measure several additional gradient directions compared to DWI, which has been used to accurately predict progression and recurrence [56]. Xiong et al. analyzed 90 samples of oligodendrogliomas (OTs) and found that both the ADC_min_ and the maximum fractional anisotropy (FA) values of DTI could distinguish the IDH status. Gliomas with IDH mutations tended to show a higher ADC_min_ (IDH-mutated = 1.11 ± 0.21; IDH-wildtype = 0.82 ± 0.13; *p* < 0.05) and a lower maximum FA (IDH-mutated = 0.23 ± 0.10; IDH-wildtype = 0.30 ± 0.07; *p* < 0.05) [57] (Table 2). Neoplastic areas often extend beyond enhanced regions, and the application of DTI enables better definition of tumor borders, showing the areas of tumor invasion. Thus, DTI parameters can be used to guide surgeries with improved patient outcomes. Aliotta et al. decomposed the diffusion tensor imaging of 41 patients with preoperative LGGs into isotropic (*p*) and anisotropic (q) components and found that IDH-wildtype LGGs demonstrated lower and fewer variable *p*-values and higher q-values, while IDH-mutated LGGs showed a wider range of *p*-values and a lower frequency of high q-values [58]. Certain studies revealed that the tumor volume shown in the *p* and q of DTI was positively correlated with the PFS of GBM patients, suggesting that surgical resection based on *p* and q could improve PFS [59].

### 3.4. Diffusion Kurtosis Imaging (DKI)

DKI is an extension of DTI, which describes the non-Gaussian distribution of water diffusion. We proposed that DKI could reflect complex water diffusion and tumor heterogeneity more precisely than DTI [60,61]. Recently, certain studies suggested that DKI was superior to DTI in the detection of microstructural changes among different glioma grades and genotypes [62,63]. In addition, DKI parameters (axial Kurtosis (Ka), radial Kurtosis (Kr), mean Kurtosis (Mk)) were useful for prognosis evaluation. Tan et al. measured 58 astrocytomas and found that Mk (IDH-mutated = 0.48 ± 0.16; IDH-wildtype = 0.67 ± 0.13; *p* < 0.001), Ka (IDH-mutated = 0.53 ± 0.17; IDH-wildtype = 0.66 ± 0.14; *p* = 0.002), and Kr (IDH-mutated = 0.45 ± 0.18; IDH-wildtype = 0.68 ± 0.19; *p* < 0.001) were significantly lower in IDH-mutated astrocytomas than in IDH-wildtype groups [64] (Table 2). Zhao et al. also compared the DKI parameters of 28 HGGs and 23 LGGs and demonstrated that the Ka, Kr, and Mk of LGGs were lower than those of HGGs [65]. Furthermore, the value of DKI in identifying IDH status was significant (*p* ≤ 0.03), and Ka (sensitivity: 74%, specificity: 75%, AUC: 0.72) had the highest diagnostic value among them [65]. The above parameters were positively correlated with Ki-67 (*p* < 0.001), indicating that a high Ki-67 value was negatively associated with glioma prognosis.

**Table 1:** IDH1mutation status of patients with gliomas (cMRI).

Variable	First Author, Year	Classification	IDH1 Status			*p*-Value
			Total	Mutant	Wildtype	
Age	Song et al. (2014) [26]	Astrocytoma	36.5	32.7	42.5	<0.001
	Xiong et al. (2016) [57]	Oligodendroglial tumor		37.9 ± 10.2	44.5 ± 12.1	0.038
	Lasocki et al. (2017) [23]	Glioblastoma		50.6 ± 11.2	64.9 ± 12.0	0.014
	Xing et al. (2017) [50]	Astrocytoma		35.76 ± 9.13	45.96 ± 18.36	0.041
	Xing et al. (2019) [49]	Glioblastoma		40.7 ± 10.77	52.23 ± 12.71	0.008
	Tan et al. (2020) [64]	Astrocytoma		43.23 ± 11.87	55.97 ± 11.74	0.001
Frontal lobe location (Yes/no) (cMRI)	Song et al. (2014) [26]	Astrocytoma	66/127	53/64	13/63	NA
	Wang et al. (2015) [33]	Anaplastic glioma	50/34	45/22	5/12	0.005
	Lasocki et al. (2017) [23]	Glioblastoma	58/95	2/3	56/92	1
	Xing et al. (2017) [50]	Astrocytoma	13/29	9/8	4/2	0.006
	Xing et al. (2019) [49]	Glioblastoma	32/43	9/1	23/42	0.002
Contrast enhancement (Yes/no) (cMRI)	Song et al. (2014) [26]	Astrocytoma	96/97	43/74	53/23	<0.001
	Wang et al. (2015) [33]	Anaplastic glioma	173/43	57/27	116/16	<0.001
	Xiong et al. (2016) [57]	Oligodendroglial tumor	43/41	38/29	5/12	0.013
	Wang et al. (2016) [34]	Glioblastoma	256/24	33/12	223/12	<0.001
	Xing et al. (2017) [50]	Astrocytoma	19/23	6/11	13/12	0.286
	Tan et al. (2020) [64]	Astrocytoma	52/10	21/9	31/1	0.002
Edema (Yes/no) (cMRI)	Song et al. (2014) [26]	Astrocytoma	84/109	46/71	38/38	0.181
	Xiong et al. (2016) [57]	Oligodendroglial tumor	57/27	47/20	10/7	0.372
	Wang et al. (2016) [34]	Glioblastoma	213/67	32/13	181/54	0.395
	Xing et al. (2017) [50]	Astrocytoma	10/32	3/14	7/18	0.746
	Tan et al. (2020) [64]	Astrocytoma	39/23	11/19	28/4	0.000
Hemorrhage (Yes/no) (cMRI)	Xiong et al. (2016) [57]	Oligodendroglial tumor	6/78	3/64	3/14	0.06
	Xing et al. (2019) [49]	Glioblastoma	58/17	9/1	49/16	0.439
	Tan et al. (2020) [64]	Astrocytoma	12/50	4/26	8/24	0.249
Necrosis (Yes/no) (cMRI)	Xiong et al. (2016) [57]	Oligodendroglial tumor	42/42	37/30	5/12	0.162
	Xing et al. (2019) [49]	Glioblastoma	62/13	8/2	54/11	1.000
	Tan et al. (2020) [64]	Astrocytoma	45/17	21/9	24/8	0.697

Notes: IDH1, isocitrate dehydrogenase 1 mutation; NA, not available.

### 3.5. Magnetic Resonance Spectroscopy (MRS)

The mechanism by which IDH mutations promote tumorigenesis has shown that 2-HG is the main signature metabolite, and the increase in 2-HG is associated with tumor cell proliferation [66]. We found that 2-HG levels could provide prognostic information for gliomas. 2-HG is normally below the sensitivity threshold of MRS (1 mM) and thus can hardly be detected, but it may become measurable due to accumulation when IDH is mutated [67]. Therefore, MRS may be a noninvasive method to detect IDH mutation status and quantitatively measure the associated increase in 2-HG and thus perform genotyping. Notably, some studies have reported that mutated IDH2 produces more 2-HG than mutated IDH1 [68]. Because of the complex spectral overlap of multiple metabolites in vivo (e.g., γ-aminobutyric acid (GABA), glutamic acid, and glutamine), 2-HG test results are often confusing, resulting in false positives [69]. Choi et al. found that the 2-HG concentration was less than 1 mM in all patients with IDH-wildtype gliomas. Therefore, 1 mM was set as the minimum threshold for the molecular diagnosis of IDH-mutated gliomas based on 2-HG MRS. Choi et al. also found that the 2-HG concentration decreased during treatment, especially in OTs [70]. This finding is consistent with that of a previous study, which analyzed 2-HG in 89 glioma patients who received radiotherapy and chemotherapy and found that the 2-HG level in the IDH-mutated genotype was gradually decreased, suggesting that the treatment was effective and that the prognosis was better [71].

### 3.6. Amide Proton Transfer (APT)

APT imaging, a specific type of chemical exchange-dependent saturation transfer (CEST) imaging [72], produces image contrast based on endogenous cellular mobile proteins in tissues [73]. APT imaging shows unique efficacy in glioma identification due to the presence of overexpressed proteins in tumors. According to multi-ROI analysis, Jiang et al. found that both the maximum APT value (0.99 ± 0.33 vs. 2.03 ± 0.72, *p* < 0.001) and the minimum APT value (0.59 ± 0.32 vs. 0.99 ± 0.47, *p* = 0.02) of IDH-mutated gliomas were significantly lower than those of IDH-wildtype gliomas. According to histogram analysis, IDH-mutated gliomas had remarkably lower mean (0.93 ± 0.44 vs. 1.39 ± 0.49; *p* = 0.03) and 50th percentile (0.96 ± 0.36 vs. 1.39 ± 0.46; *p* = 0.02) APT values than IDH-wildtype gliomas [74] (Table 2). Significant differences in APT were found among tumor grades [75]. Similar to previous studies, HGGs showed elevated APT signal intensities compared with LGGs [76]. Certain studies revealed that a high APT signal was an important predictor of poor PFS and OS in univariate analysis [77], and APT showed a particular superior prognostic value over molecular markers and clinical prognostic factors and might predict IDH mutation status.

**Table 2:** IDH1 mutation status of patients with gliomas (aMRI).

First Author, Year	Classification	Variable	IDG Status		*p*-Value
			Mutant	Wildtype	
Xiong et al. (2016) [57]	Oligodendroglial tumor	Maximal FA (DTI)	0.23 ± 0.10	0.30 ± 0.07	0.009
		Ratio of maximal FA (DTI)	0.33 ± 0.15	0.44 ± 0.11	0.004
		Oedema FA (DTI)	0.26 ± 0.14	0.21 ± 0.08	0.138
		Ratio of oedema FA (DTI)	0.37 ± 0.20	0.30 ± 0.11	0.15
		Normal FA (DTI)	0.71 ± 0.03	0.69 ± 0.03	0.122
		Minimal ADC (×10^−3^ mm^2^/s) (DWI)	1.10 ± 0.22	0.81 ± 0.16	0.001
		Ratio of minimal ADC (DWI)	1.40 ± 0.32	1.13 ± 0.23	0.002
		Oedema ADC (×10^−3^ mm^2^/s) (DWI)	1.20 ± 0.30	1.37 ± 0.30	0.036
		Ratio of oedema ADC (DWI)	1.67 ± 0.43	1.91 ± 0.42	0.034
		Normal ADC (DWI)	0.72 ± 0.03	0.72 ± 0.03	0.746
Tan et al. (2019) [64]	Astrocytoma	MK (DKI)	0.48 ± 0.16	0.67 ± 0.13	<0.001
		Kr (DKI)	0.45 ± 0.18	0.68 ± 0.19	<0.001
		Ka (DKI)	0.53 ± 0.17	0.66 ± 0.14	0.002
		MD (DKI)	1.49 ± 0.41	1.22 ± 0.26	0.005
		FA (DTI)	0.18 ± 0.17	0.20 ± 0.09	0.408
Xing et al. (2017) [50]	Astrocytoma	ADCmin (×10^−3^ mm^2^/s) (DWI)	1.21 ± 0.27	0.87 ± 0.18	<0.001
		rADC (DWI)	1.88 ± 0.41	1.37 ± 0.31	<0.001
		rCBVmax (PWI)	1.41 ± 0.50	3.47 ± 2.34	0.004
Jiang et al. (2017) [74]	Glioma	Maximum APT	0.99 ± 0.33	2.03 ± 0.72	<0.001
		Minimum APT	0.59 ± 0.32	0.99 ± 0.47	0.02
		Mean APT	0.93 ± 0.44	1.39 ± 0.49	0.03

Notes: ADC, apparent diffusion coefficient; DKI, diffusion kurtosis imaging; DTI, diffusion tension imaging; MK, mean kurtosis; Kr, radial kurtosis; Ka, axial kurtosis; MD, mean diffusivity; FA, fractional anisotropy; rCBV, relative cerebral blood volume.

### 3.7. Physiological MRI of Oxygen Metabolism

It has been demonstrated that IDH mutations could significantly reduce the expression of HIF-1α and neovascularization [46]. Physiological MRI of oxygen metabolism may contribute to the detection of IDH mutations and classification of gliomas. Stadlbauer et al. analyzed imaging biomarkers for glioma patients and found that WHO grade III and IV gliomas showed regions with decreased OEF (−54% (*p* < 0.001, n = 21), −49% (*p* < 0.001, n = 41)), while LGGs showed increased OEF (+18%, *p* < 0.001, n = 20) compared with normal tissues [78]. This allowed a clear differentiation between low- and high-grade glioma (AUC, 1), with a sensitivity of 1 and a specificity of 1 for the patient cohort. MTI had the highest diagnostic performance (AUC, 0.782; sensitivity, 0.854; specificity, 0.714) for differentiation between gliomas of grades III and IV among all biomarkers. CMRO2 was decreased (*p* = 0.037) in low-grade glioma with a mutated IDH gene, and MTI was significantly increased in glioma grade III with IDH mutation (*p* = 0.013) when compared with the IDH-wildtype counterparts. However, noninvasive assessments of neovascularization and oxygen metabolism remain challenging.

## 4. MRI-Based Radiomics Could Predict IDH Status and Clinical Outcome in Patients with Gliomas

Radiomics, an emerging discipline that aims to make predictions and obtain medical insights, is based on the extraction of quantitative features from medical images. Radiomics is an ideal complementary clinical tool because it is noninvasive and is characterized by an entire three-dimensional tumor landscape, including spatial heterogeneity [79]. Conventional MRI application is generally limited to diagnostic and postoperative evaluation, but the emerging field of radiomics has begun to expand its roles [80,81]. Several radiomics models associated with IDH1 mutation status in LGGs and HGGs have been reported [82,83,84].

Previous studies demonstrated that IDH-wildtype gliomas have more post-angiographic enhancement than IDH-mutated gliomas [34]. Liu et al. further investigated these differences by analyzing quantitative imaging features on T2WI in 158 cases of IDH mutant and wildtype WHO II/III LGGs, of which 14 imaging features were of significance for predicting IDH mutation status [85]. The radiomics model based on multiparametric MR imaging from multiregional features showed the potential for preoperative detection of IDH1 status in glioma patients. In a retrospective study, 1614 multiregional features were extracted from 225 patients. Using all relevant feature selection and random forest classification, three multiregional radiomics models were constructed to predict the status of IDH1 from the tumor core, the whole tumor, and all regions [86]. Kim et al. optimized the multiparametric MRI radiomics model using a random forest feature selector, with segmentation stability of a concordance correlation coefficient (CCC) threshold of 0.8. The multiparametric MR radiomics performance (AUC 0.795) also resembled that of the conventional radiomics model (AUC 0.729) for IDH mutation. In glioma grading, the multiparametric model with ADC features (AUC 0.932) was superior to the conventional model (AUC 0.555). Furthermore, the independent validation sets showed the same trend [87].

With the increased use of artificial intelligence in radiomics, both deep learning and machine learning can be used to classify and predict the genetic features of gliomas by fully capitalizing on all quantitative information. These features play a crucial role in therapeutic management and prognostication. Current deep learning approaches are typically convolutional neural networks (CNNs). Chang et al. trained CNNs to identify IDH status. For IDH1 mutations, the most predictive features are as follows: well-defined tumor borders, central areas of cysts with low T1 and FLAIR suppression, and minimal or absent enhancement [87]. This result is consistent with the existing literature, in which IDH-mutated tumors showed well-defined tumor borders [27] and minimal or absent enhancement [26,43]. In contrast, IDH-wildtype tumors showed thick and irregular enhancement with invasive edema. Certain studies performed preoperative imaging of LGG and HGG patients, dividing them into testing, training, and validation sets and training CNNs for each MRI sequence; through the neural network model, the prediction accuracy of IDH reached 85.7% (AUC = 0.94), 82.8% (AUC = 0.90), and 83.0% (AUC = 0.93), respectively. After factoring age into the model, the values for the testing, training, and validation sets were further improved [82]. Zhou et al. extracted 126 features (shape, histogram, and texture) from each patient’s preoperative MR imaging T1 contrast enhancement and T2-FLAIR sequences. These extracted features were then combined with age using a random forest algorithm (one of the machine learning algorithms for clinical classification) to generate a model predictive of IDH mutation status and 1p19q codeletion [88].

The value of radiomics as a prognostic factor in patients with gliomas is still under investigation. Li et al. calculated radiological features that were significantly related to OS (*p* < 0.05) and then calculated the radiomics risk score to divide LGGs into low- and high-risk groups. Multivariate Cox analyses confirmed that this risk score was an independent prognostic factor (*p* = 0.042) [85]. Peeken et al. established a combined prognostic evaluation model for pathological, clinical, and radiomics in 189 patients with GBM. This study first demonstrated the correlation between multiple semantic imaging features of gliomas and PFS and OS and proposed that the combination of pathology, clinical, and radiomics might improve the performance of the prognostic model [89].

At present, relevant radiomics studies still focus on preoperative grading and the prediction of IDH phenotypes. There are few reports on radiomics for the prognosis evaluation of gliomas with different IDH phenotypes, which can serve as a new research direction in the future to provide technical support for accurate diagnosis and individualized treatment. With the continuous supplementation of database sample size, the development of computer technology, and the establishment of more accurate and suitable mathematical models, the use of radiomics will definitely improve the clinical diagnosis of gliomas.

## 5. Conclusions

More recently, molecular biomarkers have gained importance in providing both ancillary and defining diagnostic information. Immense progress in the field of transcriptomic, genomic, and epigenetic profiling has led to the generation of new classifications and treatments for gliomas. Although surgery, alkylating agent chemotherapy, and radiotherapy are still the main treatments, individually tailored strategies based on tumor-dominant signaling pathways and antigenic tumor profiles may ultimately improve treatment outcomes. Immunohistochemistry and genomic sequence analysis are regarded as “gold standard” methods for detecting IDH mutations in patients with glioma. But neither method provides preoperative detection of IDH1 gene status. Therefore, a noninvasive and accurate method to predict IDH mutation may have great potential in routine clinical practice and could help with the implementation of appropriate management procedures in patients with glioma. Recent studies have shown that gross total resection is more beneficial for IDH-mutant gliomas than other molecular subtypes. Although maximum tumor resection is the standard treatment regardless of IDH status, the preoperative prediction of IDH status may potentially help in appropriate management procedures in patients, such as planning for treatments (including surgery). For example, neoplastic areas often extend beyond enhanced regions, and the application of DTI enables a better definition of tumor borders, showing the areas of tumor invasion. Thus, DTI parameters can be used to guide surgeries with improved patient outcomes. In addition, more aggressive and experimental treatments may be justified in patients with poor prognosis. In addition, some patients who cannot undergo surgery (such as brainstem glioma patients or patients with poor physical condition) can intervene treatment by predicting IDH status through preoperative imaging.

Results from the current study suggest that imaging features could be used to predict IDH1. Although this needs to be confirmed in a large prospective trial, these results suggest that imaging features might be able to serve as a useful biomarker of IDH1 status. For example, in cMRI, most IDH1 mutant tumors were nCET, and the frontal lobe predilection for IDH1 tumors is notable. Edema, necrosis, and hemorrhage are associated with poor prognosis. In aMRI, the rCBVmax (PWI) is significantly correlated with IDH-mutated tumors, which are significantly lower than that of IDH-wildtype. When the rADCmin (DWI) of a GBM was >0.98, it was suggestive of an IDH-mutated GBM. Gliomas with IDH mutations tended to show a higher rADC, ADCmin, and a lower maximum FA (DTI). In addition, DKI parameters (axial Kurtosis (Ka), radial Kurtosis (Kr), mean Kurtosis (Mk)) were significantly lower in IDH-mutated gliomas than in IDH-wildtype groups. The mechanism by which IDH mutations promote tumorigenesis has shown that 2-HG is the main signature metabolite. 2-HG is normally below the sensitivity threshold of MRS (1 mM) and thus can hardly be detected, but it may become measurable due to accumulation when IDH is mutated. IDH-wildtype showed elevated maximum APT and minimum APT signal intensities compared with IDH mutated gliomas. CMRO2 was decreased (*p* = 0.037) in low-grade glioma with a mutated IDH gene, and MTI was significantly increased in glioma grade III with IDH mutation (*p* = 0.013) when compared with the IDH-wildtype counterparts. Radiomics, characterized by an entire three-dimensional tumor landscape, is based on the extraction of quantitative features from medical images. Current deep learning approaches are typically convolutional neural networks (CNNs) to identify IDH status. For IDH1 mutations, the most predictive features are as follows: well-defined tumor borders, central areas of cysts with low T1 and FLAIR suppression, and minimal or absent enhancement.

MRI and MRI-based radiomics offer noninvasive and cost-effective methods. cMRI can be used to diagnose gliomas noninvasively and to evaluate prognosis by measuring the basic conditions of a glioma, such as the tumor location, contrast enhancement, and invasion of adjacent tissues. aMRI (PWI, DWI, MRS, etc.) has been widely used in the preoperative evaluation and follow-up of gliomas, providing information (angiogenesis, blood volume, micronecrosis, cellular information, etc.) that cMRI cannot provide. The combination of aMRI and cMRI could further improve diagnostic accuracy. Radiomics can extract quantitative information to improve clinical diagnosis or outcome, providing a noninvasive and powerful tool for gaining insights into pathogenesis and therapeutic responses. In general, MRI-based noninvasive techniques have incomparable advantages in predicting different types of gene mutations, predicting the PFS and OS of patients with gliomas, and evaluating the efficacy of personalized targeted therapies. Once MRI-based noninvasive techniques are sufficiently advanced, surgical tissue diagnosis by biopsy may be reserved only for a subset of MRI nondiagnostic cases. With the continuous supplementation of database sample size, the development of computer technology, and the establishment of more accurate and suitable mathematical models, the use of radiomics will definitely improve the clinical diagnosis of gliomas.
